# Validity of single item responses to short message service texts to monitor depression: an mHealth sub-study of the UK ACUDep trial

**DOI:** 10.1186/s12874-015-0054-6

**Published:** 2015-07-30

**Authors:** Ada Keding, Jan R. Böhnke, Tim J. Croudace, Stewart J. Richmond, Hugh MacPherson

**Affiliations:** 1 Department of Health Sciences, University of York, Heslington, York, YO10 5DD UK; 2 Mental Health and Addiction Research Group, Hull York Medical School, York, UK; 3 School of Nursing and Midwifery and Social Dimensions of Health Institute, University of Dundee, Dundee, UK; 4 Sydera Research Associates, Market Weighton, York, UK

**Keywords:** Validity, Text Messaging, SMS, mHealth, Depression, PHQ-9, Factor Analysis, DIF, Response Bias

## Abstract

**Background:**

An increasing number of research designs are using text messaging (SMS) as a means of self-reported symptom and outcome monitoring in a variety of long-term health conditions, including severity ratings of depressed mood. The validity of such a single item SMS score to measure latent depression is not currently known and is vital if SMS data are to inform clinical evaluation in the future.

**Methods:**

A sub-set of depressed participants in the UK ACUDep trial submitted a single SMS text score (R-SMS-DS) between 1 and 9 on how depressed they felt around the same time as completing the PHQ-9 depression questionnaire on paper at 3 months follow-up of the trial. Exploratory categorical data factor analysis (EFA) was used to ascertain the alignment of R-SMS-DS scores with the factor structure of the PHQ-9. Any response bias with regard to age or gender was assessed by differential item functioning (DIF) analysis.

**Results:**

Depression scores based on the PHQ-9 and R-SMS-DS at 3 months were available for 337 participants (74 % female; mean age: 42 years, SD = 11.1), 213 of which completed the two outcomes within 6 days of each other. R-SMS-DS scores aligned with the underlying latent depression of the PHQ-9 (factor loading of 0.656) and in particular its affective rather than somatic dimension. The R-SMS-DS score was most strongly correlated with depressed mood (*r* = 0.607), feeling bad about oneself (*r* = 0.588) and anhedonia (*r* = 0.573). R-SMS-DS responses were invariant with respect to gender (*p* = 0.302). However, there was some evidence for age related response bias (*p* = 0.031), with older participants being more likely to endorse lower R-SMS-DS scores than younger ones.

**Conclusions:**

The R-SMS-DS used in the ACUDep trial was found to be a valid measure of latent affective depression with no gender related response bias. This text message item may therefore represent a useful assessment and monitoring tool meriting evaluation in further research. For future study designs we recommend the collection of outcome data by new health technologies in combination with gold standard instruments to ensure concurrent validity.

## Background

Depression is a debilitating long-term health condition that is one of the leading causes of global disease burden [1, 2], and its management presents a major challenge to health care providers worldwide. As part of an emerging trend to utilise mobile devices in health care (mHealth) [3], ubiquitous mobile technologies such as short message service (SMS or text messaging) may offer a cheap and straightforward support tool to monitor outcomes in clinical care and self-management of depression and other chronic health conditions [4]. Text messaging has already been studied in the management of diabetes [5–7], asthma [8–10], lower back pain [11–13] and irritable bowel syndrome [14] for example, as well as in the support of long-term health behaviour change interventions such as weight loss [15, 16] and smoking cessation [17]. While the importance of validating health outcomes collected by text messaging has been recognised, few of the studies using SMS technology have implemented this [18, 19].

Within mental health, research has primarily focussed on utilising text messaging for the management of bipolar disorder and schizophrenia. Feasible symptom monitoring was demonstrated when gathering weekly responses of validated questionnaires for depression and mania from bipolar patients [20] and when collecting daily outcomes on several symptom dimensions from patients suffering from schizophrenia [21]. Furthermore, when employed as a low level intervention in schizophrenia, customised daily text prompts for different illness aspects improved outcomes in those areas [22], and weekly monitoring of early warning signs by patients and relatives improved rates of relapse and hospital readmission [23].

Until recently, only a small number of studies with few participants had looked specifically at the possibility of collecting depression outcomes by text message. A single item SMS subjective distress rating (scale 0 to 10) was used for daily mood monitoring in patients with anxiety or depression in a remote Australian community during and after treatment [24], and a daily SMS mood score (scale 1 to 9) was collected as an adjunct to cognitive behavioural therapy (CBT) for outpatients from different ethnic groups in the United States [25–27]. These studies found mood data collection by SMS feasible, acceptable, and predictive of PHQ-9 [28] depression scores. This has been further confirmed in a sub-study of the UK ACUDep trial [29], which collected weekly depression scores (scale 1 to 9) by text message from over 500 depressed adult participants during the first 3 months of trial follow-up [30]. The study demonstrated good response rates (94 % of patients responded to at least one text prompt, and patients replied to an average of 12.5 (SD = 3.45) of 15 texts), the depression rating correlated well with the PHQ-9 measure of depression (Kendall’s tau-b = 0.570), and SMS depression scores were sensitive to change in response to the trial treatments.

Monitoring patient depression with such a simple, single SMS text score instead of the administration of lengthy questionnaires represents an attractive mode of data collection in view of compliance rates and patient burden. This is in line with other efforts to condense the measurement of depression into one or two items for the purpose of efficient patient screening and monitoring [31–34]. The choice between long and short form assessment tools will depend on the context and purpose of the evaluation, balancing ease of data collection with the need for robust clinical diagnoses [35]. It remains unknown whether a single SMS depression score, as used in the ACUDep trial, can be considered a valid measure of depression and could consequently be recommended for use in research and evaluation in clinical practice.

The present study therefore aimed to establish the validity of the ACUDep SMS depression score (termed R-SMS-DS [30]), by employing item response theory methodologies. If scores obtained for the R-SMS-DS and the PHQ-9 both measure the same latent depression variable, then this could be confirmed by including all individual items in a factor analysis. The PHQ-9 has variously been shown to be either uni-dimensional in primary care patients [36–39], or to divide into an affective and somatic dimension in certain patient populations [40–43]. It was of interest whether R-SMS-DS scores would align with either one of these dimensions if present in the ACUDep patient sample.

Depression prevalence, symptomatology and trajectories are known to differ between men and women [44–46] as well as over the course of life [45, 47]. Although the reasons for these disparities remain debated, they may be connected to differential use of health care systems [48] and important aspects of depression treatment [49]. It is therefore important that these demographic groups do not differ in the way they use the R-SMS-DS, and score differences between individuals only reflect variations in their respective levels of depression [50]. Therefore the present study also aimed to assess any response bias for the R-SMS-DS with respect to age and gender. The absence or presence of such biases will provide evidence for the relative impact of these factors on the measurement of depression with the R-SMS-DS, before it can be considered to inform valid treatment decisions in clinical practice.

Results of this study were anticipated to inform recommendations for whether and how the increasing number of research studies using mHealth technologies for patient monitoring should incorporate these tools and their validation into their study designs.

## Methods

### Participants

Participants included in this study took part in the ACUDep trial [29], a three arm randomised controlled trial that evaluated the effectiveness of acupuncture or counselling compared to standard care in a population of depressed adults in the North of England. Participants were 18 years of age or older, had consulted for depression within the previous five years and had ongoing depression with a score of 20 or above on the Beck Depression Inventory (BDI-II) [51]. Those recruited into the trial were invited to take part in an optional sub-study involving the use of weekly SMS text messages to monitor their depression. 755 patients were recruited into the ACUDep trial between 2009 and 2011, and 527 of these consented to the SMS sub-study.

### Design

In order to investigate the validity of the R-SMS-DS [30] as a measure of depression, this study exploited the collection of the last of 15 weekly SMS text scores and PHQ-9 depression by questionnaire around the same time at 3 months follow-up of the trial. Participants were considered as a single patient group for this purpose, irrespective of their allocated trial arm. The differences in R-SMS-DS scores between treatment groups in patients’ depression trajectories are reported elsewhere [30]. We used categorical data factor analysis [52] to ascertain the factor structure of the PHQ-9 in the present patient sample and the alignment of the R-SMS-DS with that structure. Following these exploratory analyses we used differential item functioning (DIF) analysis to investigate potential response bias with respect to age or gender.

### Outcome measures

**Fig. 1:**
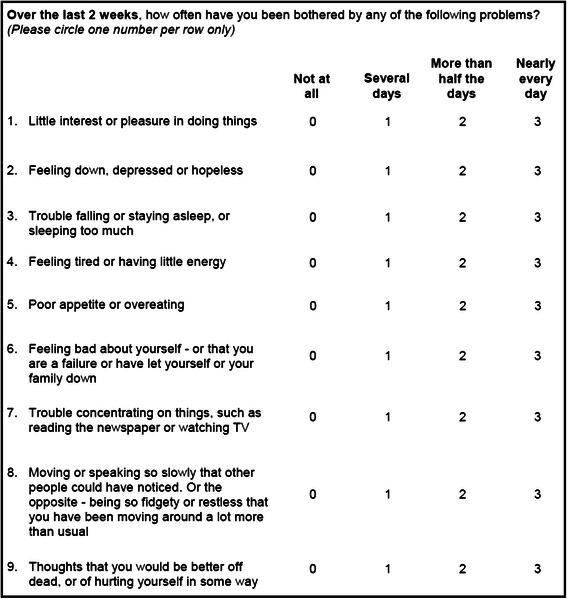
PHQ-9 Questionnaire Wording (Source: Kroenke et al. 2001)

The PHQ-9 [28] is a nine-item depression scale based on the DSM-IV symptom criteria for major depressive disorder [53]. It is used routinely as a screening tool in clinical practice and as a standard depression severity outcome in research. Each item is scored between 0 and 3, thus PHQ-9 total scores range from 0 to 27 with higher scores indicating greater depression (see Fig. 1 for complete wording of the PHQ-9). The instrument was completed by patients at baseline and follow-up on paper questionnaires, and the total score at 3 months served as the ACUDep primary endpoint.

The weekly R-SMS-DS text message sent to patients who consented to the sub-study contained the text: ‘ACUDep Trial: Over the last week how depressed have you felt on average? Please reply with a score between 1 and 9; where 1 is “not at all” and 9 is “extremely”’. Up to 15 weekly text messages were sent to participants following randomisation, the final text approximately coinciding with 3 months follow-up. Received participant texts were matched to the text they were responding to, and text content was validated to arrive at a single score for each responding patient between 1 and 9, allowing half scores if patients submitted these or two adjacent scores.

### Statistical analysis

Exploratory Factor Analyses (EFAs) were conducted for three groupings of ACUDep participants at 3 months follow-up: Group 1 comprised patients with complete PHQ-9 items; Group 2 were patients with complete PHQ-9 items and a valid R-SMS-DS score; and Group 3 were patients with complete PHQ-9 items and a valid R-SMS-DS score completed within 6 days of each other. Previous research suggests that PHQ-9 scores are associated with average texted mood ratings over 1 week, but not 2 weeks [27]. Group 1 was used to inform the factor structure of the PHQ-9, whereas the alignment of R-SMS-DS scores with PHQ-9 depression was explored in Groups 2 and 3, with greater agreement expected in the temporally closer assessments in Group 3.

All EFAs were computed using FACTOR 9.2 [54], using polychoric correlations in a parallel, minimum rank factor analysis with oblique (promin) rotation. One- and two-factor solutions were implemented as suggested by previous structural analyses of the PHQ-9 [36–43]. Optimal dimensionality of the item set was established, for which parallel analysis has been shown to be highly efficient [55–57]. It determines eigenvalues for random data matrices and establishes a cut-off (above 95 % based on random data) to retain relevant factors only, i.e. those that capture more common variance between the items than expected purely by chance. Item correlations between all item pairs were extracted from the analyses as well as factor loadings for the one- and two-factor models, suppressing any loadings less than 0.400. Emphasis of these analyses was on the fit of the R-SMS-DS score with the PHQ-9 factor structure.

Differential Item Functioning (DIF) with respect to age and gender was investigated by ordinal logistic regression [58, 59] in Stata version 12 [60]. The analyses included all patients with complete PHQ-9 and R-SMS-DS data at 3 months (Group 2), predicting R-SMS-DS score (values 1 to 9) from age or gender (uniform DIF) and their interaction with the PHQ-9 (non-uniform DIF, i.e. any bias that was dependent on the level of latent depression). The regression models controlled for latent depression as measured by the total PHQ-9 score at 3 months follow-up, which was expected to be highly correlated with the R-SMS-DS score, reflecting that both assess the same underlying depression construct. Evidence for response bias would be found if age, gender or their interactions with the PHQ-9 significantly (*p* < 0.05) predicted the R-SMS-DS over and above the PHQ-9 total score, potentially rendering comparisons between them unfair [50]. The direction of any identified DIF was explored, and the DIF effect size determined by comparison of pseudo R^2^ values between the analysis models and a base model including PHQ-9 total score as the only predictor. Continuous variables (age and PHQ-9) were centred for all analyses.

### Ethical approval and consent

Full ethical approval for the trial was granted by York NHS Research Ethics Committee on 21st September 2009 (ref: 09/H1311/75), together with research governance approval shortly thereafter from North Yorkshire & York Primary Care Trust. All participants provided informed written consent.

## Results

### Data availability and baseline characteristics

Of 755 randomised ACUDep trial participants, 602 patients had complete PHQ-9 data for all items at 3 months follow-up (Group 1). Of the 527 ACUDep participants who additionally consented to take part in the SMS sub-study, 373 patients responded with a valid text message to their last follow-up SMS, which broadly coincided with the 3-month PHQ-9 follow-up time point. Of these, 337 had complete PHQ-9 data (Group 2). PHQ-9 questionnaires were completed on average 8 days from responding to the R-SMS-DS (range −8 to 75 days, completion date missing for 11 patients), and 213 patients (63 %) completed these outcomes within 6 days (Group 3). Baseline characteristics for all randomised ACUDep patients and the different patient groups included in the factor analyses are given in Table 1. Apart from fewer retired patients in Group 3, the demographic profile did not substantially differ between groups.

**Table 1:** Baseline characteristics of different analysis populations

Characteristic	Total patients in ACUDep trial *n* = 755	Group 1 Patients with PHQ-9 score at 3 months *n* = 602	Group 2 Patients with PHQ-9 score at 3 months & R-SMS-DS at 3 months (any time) *n* = 337	Group 3 Patients with PHQ-9 score at 3 months & R-SMS-DS at 3 months (±6 days) *n* = 213
Age				
Mean (SD)	43.5 (13.37)	44.7 (13.14)	42.2 (11.13)	42.5 (11.18)
Median (min, max)	43 (18, 93)	43.5 (18, 89)	42 (18, 75)	42 (18, 75)
Gender, n (%)				
Male	201 (26.6)	159 (26.4)	86 (25.5)	50 (23.5)
Female	554 (73.4)	443 (73.6)	251 (74.5)	163 (76.5)
Employment, n (%)				
Working full-time	281 (37.2)	223 (37.0)	140 (41.5)	80 (37.6)
Working part-time	144 (19.1)	116 (19.3)	63 (18.7)	47 (22.1)
Unable to work	95 (12.6)	69 (11.5)	36 (10.7)	20 (9.4)
Looking after home	83 (11.0)	62 (10.3)	37 (11.0)	25 (11.7)
Retired	65 (8.6)	61 (10.1)	15 (4.5)	10 (4.7)
Full-time education	23 (3.0)	17 (2.8)	13 (3.9)	8 (3.8)
Other	48 (6.4)	40 (6.6)	23 (6.8)	18 (8.5)
Missing	16 (2.1)	14 (2.3)	10 (3.0)	5 (2.3)
Depression, mean (SD)				
Age at 1^st^ major episode	25.2 (12.28)	25.6 (12.51)	23.8 (11.03)	24.9 (11.66)
Baseline BDI-II	32.5 (8.72)	32.1 (8.62)	31.8 (8.54)	31.5 (8.27)
Baseline PHQ-9	16.0 (5.29)	15.7 (5.32)	15.6 (5.48)	15.4 (5.29)

### Factor analyses

Results of all factor analyses are presented in Table 2. The initial EFA of the PHQ-9 using all available data (Group 1, *n* = 602) confirmed the uni-dimensional structure of the scale, with the first identified factor explaining 64 % of the variance and being the only one that captured more common variance than expected by chance (parallel analysis). Individual item loadings were high and ranged between 0.704 and 0.856. When forced into a two-factor solution, the PHQ-9 items divided into two highly correlated (0.834) dimensions consistent with previous findings: a factor of somatic symptoms (sleep, fatigue, appetite) and a factor of affective symptoms represented by the remaining six depression items.

**Table 2:** Summary of exploratory factor analysis item factor loadings^a^ of PHQ-9 and R-SMS-DS scores at 3 months follow-up

	Group 1			Group 2			Group 3		
	*N* = 602 patients with PHQ-9			*N* = 337 patients with PHQ-9 & R-SMS-DS (any time)			*N* = 213 patients with PHQ-9 & R-SMS-DS (within 6 days)		
	Loadings			Loadings			Loadings		
	One-Factor	Affective	Somatic	One-Factor	Affective	Somatic	One-Factor	Affective	Somatic
PHQ-9 Item Descriptive/Variance explained	64 %	--- 73 % ---		61 %	--- 69 % ---		61 %	--- 70 % ---	
1. Loss of interest (anhedonia)	.852	.632	-	.855	.540	-	.854	.448	.453
2. Depressed mood	.856	.875	-	.876	.768	-	.882	.683	-
3. Sleep disturbance	.778	-	.942	.761	-	.973	.740	-	.994
4. Fatigue	.794	-	1.078	.794	-	1.041	.779	-	.831
5. Appetite changes	.718	-	.640	.700	-	.617	.719	-	.573
6. Feeling bad about oneself	.816	.972	-	.840	1.244	-	.846	1.169	-
7. Concentration difficulties	.791	.554	-	.738	.411	-	.746	-	.634
8. Psychomotor disturbance	.735	.507	-	.690	.405	-	.700	-	.714
9. Thoughts of death or self-harm	.704	.894	-	.724	.860	-	.719	.826	-
R-SMS-DS: ‘How depressed have you felt?’	n/a^b^	n/a^b^	n/a^b^	.656	.501	-	.692	.616	-
Correlation between factors		--- .834 ---			--- .820 ---			---.793 ---	

^a^Loadings < 0.400 suppressed

^b^Group 1 analyses excluded the SMS score, as this was not available for all patients

When including the R-SMS-DS score in the analyses (Table 3), the PHQ-9 items that correlated most strongly for any patients with both outcomes (Group 2) were depressed mood (0.607), feeling bad about oneself (0.588) and anhedonia (0.573). Correlations for the sub-set of patients whose R-SMS-DS and PHQ-9 responses were given within 6 days (Group 3) exhibited a similar pattern and were generally higher, with the exception of sleep and psychomotor disturbance. These mainly somatic depression symptoms correlated more strongly with the R-SMS-DS score when assessments were more widely spaced in time (see Table 3).

**Table 3:** Polychoric correlations between R-SMS-DS score and PHQ-9 items

PHQ-9 items	Group 2	Group 3	Grp 2 - Grp 3
	Patients with PHQ-9 & R-SMS-DS (any time) *N* = 337	Patients with PHQ-9 & R-SMS-DS (within 6 days) *N* = 213	Patients with PHQ-9 & R-SMS-DS (outside 6 days) *N* = 113
1. Loss of interest (anhedonia)	.573	.593	.545
2. Depressed mood	.607	.619	.561
3. Sleep disturbance	.474	.458	.517
4. Fatigue	.479	.487	.481
5. Appetite changes	.472	.513	.425
6. Feeling bad about oneself	.588	.665	.454
7. Concentration difficulties	.450	.485	.414
8. Psychomotor disturbance	.421	.404	.473
9. Thoughts of death/self-harm	.436	.472	.381

When R-SMS-DS scores were included in the factor analyses (Table 2), the one-factor structure remained the optimal description of the data (parallel analysis; 61 % explained variance). The R-SMS-DS text score loaded moderately highly onto the underlying depression factor: 0.656 in the overall model (Group 2) and 0.692 for texts within 6 days of PHQ-9 completion (Group 3). When analysed as a two-factor solution, the R-SMS-DS score aligned with the six items of the PHQ-9 affective dimension (0.501 for Group 2 patients). The two-factor structure altered slightly when using the sample of patients who responded within 6 days (Group 3): PHQ-9 items for concentration difficulties and psychomotor disturbance now loaded predominantly onto the somatic dimension, and anhedonia loaded equally onto the affective and somatic dimension. The R-SMS-DS score still aligned with the dimension made up of the remaining core affective items (0.616), comprising depressed mood, feeling bad about oneself and having thoughts of dying or self-harm. The two dimensions remained highly correlated however (0.793), and the parallel analysis identified a one-factor solution as optimal in this sample too, explaining 61 % of the variance.

In summary, the R-SMS-DS was shown to pick up on the same underlying depression as the PHQ-9, in particular the affective dimension of depression.

### Response bias

Following results of the EFAs, the specified PHQ-9 total score in the logistic DIF regressions was replaced with the affective sub-score PHQ-9_A_, calculated as the sum of the PHQ-9 affective items (Items 1,2,6,7,8,9). Although according to the results of the parallel analysis a one factor solution described the responses to all items, we used the PHQ-9_A_ as a measure with maximum uni-dimensionality, thereby providing a more concise estimate of the characteristic being measured by the R-SMS-DS than the total score. The resulting regression coefficients were expressed as odds ratios and are presented in Table 4.

**Table 4:** DIF ordinal logistic regression results (Group 2, *n* = 337)

Predictor	Odds ratio	SE	95 % CI	*p*
Age DIF analysis				
Age	0.98	0.009	0.96, 1.00	.031
Age x PHQ-9_A_	1.00	0.002	1.00, 1.01	.271
PHQ-9_A_	1.46	0.044	1.38, 1.55	<.001
Gender DIF analysis				
Gender (being female)	1.26	0.280	0.81, 1.95	.302
Gender x PHQ-9_A_	1.07	0.061	0.95, 1.19	.250
PHQ-9_A_	1.39	0.073	1.25, 1.54	<.001

PHQ-9_A_ = Sum of affective PHQ-9 items (Items 1,2,6,7,8,9)

The DIF analysis for age revealed no evidence for non-uniform DIF (*p* = 0.271) but some evidence for uniform age related DIF (*p* = 0.031), change in pseudo R^2^ = 0.004. Using predicted endorsements of each R-SMS-DS value based on the regression model, we found older participants being more likely to use lower scores in their text responses (R-SMS-DS scores of 1 to 3) and less likely to use higher scores (R-SMS-DS scores of 5 to 9) compared to younger participants with the same level of affective depression (PHQ-9_A_). The DIF analysis for gender revealed no evidence for uniform DIF (*p* = 0.302) nor non-uniform DIF (*p* = 0.250), change in pseudo R^2^ = 0.002. Thus results of the DIF analyses suggest some evidence of age related response bias but not gender bias for the R-SMS-DS.

## Discussion

The present study set out to validate a single depression rating item submitted by SMS text message (R-SMS-DS) against data of the widely validated PHQ-9 concurrently collected by post, which were available for a depressed adult sub-population of the UK ACUDep trial. R-SMS-DS scores were found to correlate well with latent depression when included in a combined single-factor solution explanatory factor analysis with the individual PHQ-9 items. The most closely associated PHQ-9 items were the two core DSM-IV criteria of depressed mood and anhedonia as well as feeling bad about oneself. The correlations closely mirrored those observed for a single-item paper based depression severity rating when correlated with DSM-IV criteria in a population of psychiatric outpatients undergoing treatment for major depression [32]. With the exception of sleep and psychomotor disturbances, item correlations were larger when patients completed the two assessments closer in time, therefore results suggest that the R-SMS-DS score did indeed measure depression as desired.

While the optimal one-factor model in this study lent further support to the uni-dimensionality of the PHQ-9, it was unsurprising to find that R-SMS-DS ratings aligned with the affective rather than somatic dimension of depression in the pre-specified two-factor analyses. This raises the possibility of complementing the R-SMS-DS with one or more physical symptom questions if monitoring of the somatic depression dimension is additionally desired. Sleep, fatigue and appetite were picked up as core somatic symptoms in line with all previous studies of a two-dimensional PHQ-9 structure. Interestingly, a model with these three symptoms alone forming the somatic dimension (found in selected previous research [40, 42, 61]) was supported in patients who had both valid PHQ-9 data and patients with valid PHQ-9 and any R-SMS-DS data; whereas the most commonly observed two-factor structure [40, 41, 43, 62] with the additional two somatic items of concentration difficulties and psychomotor disturbance was only observed in the sub-set of patients whose PHQ-9 and R-SMS-DS responses were closer in time (within 6 days). The possible loading of anhedonia on the somatic dimension for these patients had previously only been recorded in one study of spinal cord injury patients at a single long-term follow-up point [40]. Patient characteristics in terms of demographics and baseline depression did not appear to differ for patients in this group, so it may be the result of differences in other patient characteristics, such as present comorbidities affecting the rating of somatic symptoms. Alternatively the model factors may be less stable in this group as the smallest analysed sub-sample.

Consistent use of the R-SMS-DS was demonstrated across men and women. However, older patients were found to be less likely to endorse higher scores even when their degree of latent depression (as defined by the PHQ-9) was indicative of such an elevated level. This could be a result of a different understanding of the ‘feeling depressed’ terminology used in the text message, which has been discussed in the epidemiological literature of depression both as a shift towards a more somatically driven concept or as confounding with other somatic morbidities [63, 64]. Further reasons could be different attitudes towards communicating mental wellbeing by mobile technologies or a greater reluctance to potentially arouse cause for concern. Such age bias could affect the sensitivity of the R-SMS-DS score if used for depression screening, however it is unlikely for that to be its primary use. We envisage the R-SMS-DS as a monitoring tool for patients who have already undergone formal depression assessment. The direction of the age bias was opposite to that identified in a sample of UK primary care patients for the PHQ-9 items of low mood and anhedonia for patients aged 55 and over [65]. It remains possible that the observed bias in this study is a consequence of the relatively small total sample size or the small number of older patients in the sample. While we used age as a continuous predictor, the number of patients for whom the effect was identified based on marginal effect plots was rather low (*n* = 8 participants ≥ 65 years, 2.4 %). Moreover, the magnitude of the association between age and R-SMS-DS score (OR = 0.98) was only weak [66], and the effect size in terms of pseudo R^2^ [67] was negligible. The stability of this bias remains to be confirmed in a larger patient sample including a qualitative assessment of possible reasons.

Overall, results of this study add further support to the validity of collecting depression severity outcomes by SMS, which had already been shown to be feasible and acceptable in adults with ongoing depression in primary care in the ACUDep trial [30]. To our knowledge, this is the first study aiming to validate an SMS self-report tool for depression using item-response theory methodologies, and results are strengthened by the use of a gold standard validated patient self-report depression instrument (PHQ-9) based on DSM-IV criteria for comparison. Despite the relatively small sample size of this study, patients agreeing to submit weekly text messages and who were included in the present analyses were representative of those taking part in the ACUDep trial (Table 1), who in turn were typical of adults in the UK with ongoing depression in primary care.

However, findings cannot be extrapolated to patients who are presenting with depression for the first time or who do not consult in primary care at all. A further limitation includes the temporal difference between PHQ-9 and R-SMS-DS data completion, which had not been designed to be collected concurrently, resulting in considerable between-patient variability in the time between completing the assessments. In addition, the reference time frame differed for the two measures (PHQ-9: over the last two weeks; R-SMS-DS: average over the last week), therefore it is not certain whether patients were in the same mental state when reporting those outcomes. Indeed the positive findings of this study may only represent a conservative estimate of the level of association. However, the depression outcomes linked with one another in this study were patient reported only, and no independent assessment was carried out in order to confirm clinical validity. Moreover, only the association between R-SMS-DS and a single screening tool (PHQ-9) has been demonstrated so far, and further convergent validity needs be shown in order to establish the R-SMS-DS as a valid estimate of latent depression. Capturing the full multi-faceted nature of depression will never be possible by a single item, and this is not the aim of the R-SMS-DS monitoring tool.

For future studies we suggest to include at least one assessment that allows researchers to test the concurrent validity of their novel electronic or mHealth tools with a gold standard instrument collected at the same time, an approach that has not yet been widely adopted. The shortcomings of this study could be addressed by a more controlled, dedicated design, either as standalone work or embedded in larger investigations, with particular attention to the magnitude and context of any response bias. The successful use of tools from the framework of item response theory for the validation of SMS scores at a single time point might also be extended to investigate the longitudinal validity of the R-SMS-DS scores, which had been collected weekly over 3 months. Notwithstanding such further methodological work, we believe that findings from the present and a previous study [30] have provided sufficient evidence for the feasibility, acceptability and validity of the R-SMS-DS for monitoring depression in the ACUDep study population. Given these findings, we encourage investigators and clinicians to incorporate the R-SMS-DS as a free to use outcome measure in the study of depression management in different clinical populations. If verified against other validated depression measures and found acceptable in different clinical contexts, the R-SMS-DS could be considered for use in routine clinical practice.

## Conclusions

This study has demonstrated that the self-report R-SMS-DS depression item used in the ACUDep trial was a valid measure of the affective dimension of depression in this study population. In agreement with previous findings, the R-SMS-DS may therefore represent a useful assessment and monitoring tool meriting evaluation in further research.
